# Measuring the Impacts of Water Safety Plans in the Asia-Pacific Region

**DOI:** 10.3390/ijerph15061223

**Published:** 2018-06-10

**Authors:** Emily Kumpel, Caroline Delaire, Rachel Peletz, Joyce Kisiangani, Angella Rinehold, Jennifer De France, David Sutherland, Ranjiv Khush

**Affiliations:** 1 Department of Civil and Environmental Engineering, University of Massachusetts, Amherst, MA 01003, USA; ekumpel@umass.edu; 2 The Aquaya Institute, P.O. Box 21862-00505, Nairobi, Kenya; joyce@aquaya.org; 3 The Aquaya Institute, P.O. Box 5502, Santa Cruz, CA 95063, USA; rachel@aquaya.org (R.P.); ranjiv@aquaya.org (R.K.); 4 World Health Organization, 1211 Geneva, Switzerland; angella27@gmail.com (A.R.); defrancej@who.int (J.D.F.); 5 World Health Organization, Regional Office for South East Asia, New Delhi 110002, India; dcsuth@gmail.com

**Keywords:** water safety plans, drinking water quality, risk management, impact assessment, Asia-Pacific region

## Abstract

This study investigated the effectiveness of Water Safety Plans (WSP) implemented in 99 water supply systems across 12 countries in the Asia-Pacific region. An impact assessment methodology including 36 indicators was developed based on a conceptual framework proposed by the Center for Disease Control (CDC) and before/after data were collected between November 2014 and June 2016. WSPs were associated with infrastructure improvements at the vast majority (82) of participating sites and to increased financial support at 37 sites. In addition, significant changes were observed in operations and management practices, number of water safety-related meetings, unaccounted-for water, water quality testing activities, and monitoring of consumer satisfaction. However, the study also revealed challenges in the implementation of WSPs, including financial constraints and insufficient capacity. Finally, this study provided an opportunity to test the impact assessment methodology itself, and a series of recommendations are made to improve the approach (indicators, study design, data collection methods) for evaluating WSPs.

## 1. Introduction

Diarrheal diseases resulting from inadequate drinking water are estimated to cause 502,000 deaths per year [1]. Providing safe drinking water is essential to prevent water-related diseases. However, monitoring and maintaining water safety in piped systems and point sources around the world is challenging [2,3,4]. In 2004, the World Health Organization (WHO) formally introduced Water Safety Plans (WSPs) as a preferred management approach for ensuring the safety of drinking water supply [5]. WSPs provide a comprehensive methodology to assess and mitigate risks in all steps of the water supply system from catchment to consumer. Specifically, the approach includes the formation of a dedicated WSP team; a system assessment phase during which risks, existing control measures, and gaps are identified to inform the development and implementation of an improvement plan; routine monitoring and evaluation; and periodic review of the entire process (Figure S1). This methodology is applicable to both large piped systems and small community water supplies [6,7]. WSPs are considered “the most effective means of consistently ensuring the safety of a drinking-water supply” [8] and have been implemented in at least 93 countries [9]. However, despite growing literature on WSPs, there is no established and validated process to evaluate WSP impacts, and robust scientific evidence for the benefits of WSPs is limited.

A number of studies have documented the benefits of WSPs and the lessons learned from their implementation [10,11,12,13,14,15,16]. However, as shown in a recent literature review, there are very few examples of rigorous systematic assessments of WSP impacts [17]. Two studies in Iceland, France, and Spain assessed the outcomes of WSPs using before/after comparisons [18,19], but they included only seven and five utilities, respectively, and focused on developed countries. The US Center for Disease Control and Prevention (CDC) proposed a conceptual framework to evaluate the impacts of WSPs [19], which distinguishes shorter-term system-level changes (“outcomes”) from longer-term service delivery and societal improvements (“impacts”) and provides a comprehensive evaluation lens covering institutional, operational, financial, policy, water supply, health, and socioeconomic aspects. However, the CDC conceptual framework has not been comprehensively operationalized through field-testing of specific indicators and data collection methods. Validating an operationalized form of the CDC evaluation framework across a wide range of contexts (urban/rural, large/small systems, etc.) and geographic settings remains to be done.

Establishing a process to evaluate the effectiveness of WSPs is important in order to (1) compare WSPs with more targeted (i.e., less comprehensive) interventions to improve safe water supply, such as infrastructure upgrades, regulatory reforms, or capacity building; (2) strengthen support for WSPs among governments and sector stakeholders and (3) help strengthen WSP implementation through better understanding of the practices that are most challenging to change. At the water system level, an evaluation process can also be useful to managers for regular monitoring of WSP progress and evidence-based decision-making.

This study developed and tested methods for assessing the effectiveness of WSPs in 12 countries in the Asia-Pacific region. Specifically, it allowed for the investigation of the three following questions: (1) What types of data can be collected to evaluate WSP impacts? (2) How should these data be collected? (3) What study design should be applied depending on the context and objective of the evaluation? Using an operational impact assessment framework that was developed based on the CDC conceptual framework [20], before/after data were collected on 36 performance indicators along with qualitative information at 99 sites between November 2014 and June 2016. After presenting findings on WSP outcomes and impacts, this paper draws on the data obtained and the collection process to reflect on the challenges in evaluating WSPs and recommends study designs for future impact assessments.

## 2. Materials and Methods

### 2.1. Study Sites

**Figure 1:**
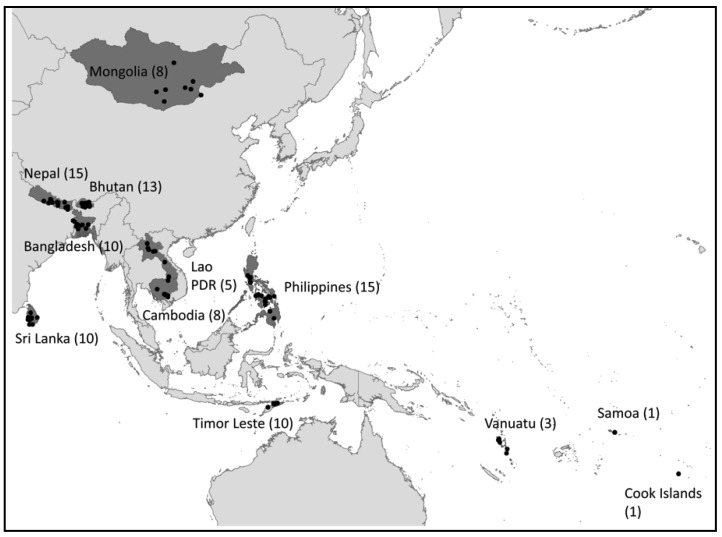
Twelve countries that participated in the WSP impact assessment, with number of participating sites indicated between parentheses.

This impact assessment was undertaken through the Water Quality Partnership for Health (WQP), a program launched in 2005 by the Australian Department of Foreign Affairs and Trade (DFAT) and WHO to introduce and institutionalize WSPs in the Asia-Pacific region. Sixteen countries receiving WQP support were invited by WHO Regional Offices to participate in the impact assessment. Among these countries, 12 were able to collect both baseline and follow-up data: Bangladesh, Bhutan, Cambodia, Cook Islands, Lao PDR, Mongolia, Nepal, Philippines, Samoa, Sri Lanka, Timor-Leste, and Vanuatu (Figure 1).

Overall, 99 sites participated in the study (Figure 1). Institutions responsible for selecting study sites varied between countries and included national-level government agencies (e.g., departments/ministries of health and water), national water suppliers, heads of local government units, and WHO country or regional staff. Site selection was non-random. Selection criteria varied between countries (Table S1), but generally reflected an attempt to capture the diversity of settings (according to system size, management type, geography, performance, challenges, etc.), while also prioritizing sites with longer-running WSPs and readily available data.

**Figure 2:**
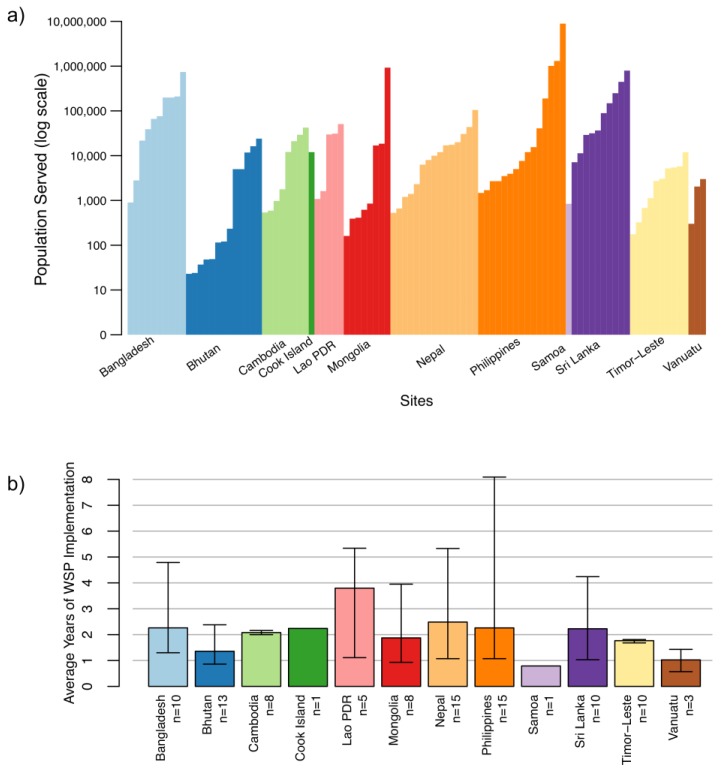
Description of sites: (**a**) Populations served by sites; (**b**) Mean age of WSP at time of follow-up data collection. The upper and lower bounds show the maximum and minimum ages in each country, respectively.

Piped systems represented 90% of the study sites (89 out of 99), with approximately two-thirds (58) of these operated by public/private utilities or local government units, and a third (31) managed by communities (Table 1). Point sources, representing 10% of the study sites (10 out of 99), included water-refilling stations and groundwater sources (Table 1). In most countries (all except Sri Lanka, Cook Islands, Samoa, and Vanuatu), sites represented a mix of rural and urban settings. Overall, 62% of the sites (61) were urban and 38% (38) were rural (Table 1). The populations served by the water systems varied widely between 22 people (a small community-managed piped system in Bhutan) to 8.9 million people (a large urban utility in the Philippines) (Figure 2a). The median population served across all the sites was 7140 people. Eight of the 10 smallest systems were in Bhutan; the 10 largest systems were in the Philippines, Mongolia, Sri Lanka, and Bangladesh (Figure 2a).

The age of WSPs implemented in the study sites varied considerably within and between countries (Figure 2b). At follow-up, the youngest WSP was 7 months (in Vanuatu) and the oldest was 8 years and 1 month (in the Philippines), with an overall mean WSP age of 25.2 months (Figure 2b).

### 2.2. Evaluation Framework

**Figure 3:**
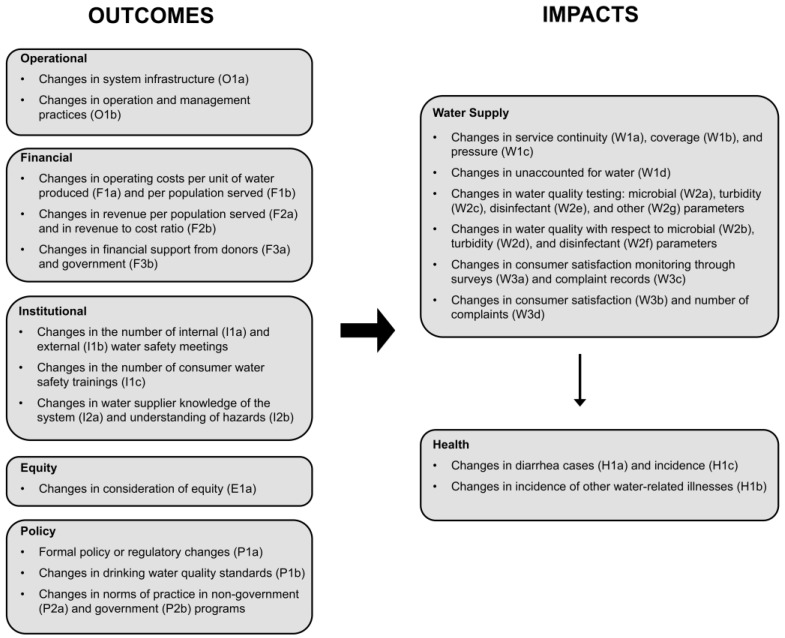
Operational impact assessment framework and indicators used in this study. More details on indicators, including units and sub-indicators, are presented in Table 2.

WHO developed an operational impact assessment framework for this study based on the CDC’s conceptual framework [19], which distinguishes between shorter-term (occurring on the order of months) system-level changes, called “outcomes”, and longer-term (occurring on the order of years) service delivery and societal improvements, called “impacts” (Figure 3). Outcomes explored in the study include changes in communication and knowledge (institutional outcomes); infrastructure and operation/management procedures (operational outcomes); revenue collection, cost recovery, and investment (financial outcomes); norms and regulations (policy outcomes); and inclusion of disadvantaged groups (equity outcomes, which were added to the CDC conceptual framework) (Figure 3). Impacts include changes in water service delivery such as coverage, continuity, quality, and potential health impacts (e.g., reduction in waterborne illness) (Figure 3). Eventual socioeconomic changes (e.g., reductions in medical expenses and coping costs) included in the CDC conceptual framework were not considered in this study due to concerns related to the practicality of data collection and because these impacts are expected to occur over longer time scales.

The operational impact assessment framework used for this study comprised 36 performance indicators, listed in Table 2, defined to measure the outcomes and impacts of WSPs. These indicators were developed in consultation with WSP stakeholders in participating countries in 2013, prior to the publication of the CDC indicators presented in Lockhart et al. [21]. Therefore, the two sets of indicators, although similar, are not identical. The operational impact assessment framework also included open-ended questions to help interpret the quantitative indicator data and gather additional information on the challenges of WSP implementation.

### 2.3. Study Design and Data Collection

An uncontrolled before-after study design was applied. Baseline data were collected between November 2014 and February 2016 and were intended to capture conditions at a site prior to WSP implementation. At 65% of sites, baseline data were collected retrospectively, i.e., after the start of the WSP. Follow-up data were collected between December 2015 and June 2016 (Figure S2, Table S2).

Data collection was undertaken by various organizations, including government agencies, national water utilities, universities, WHO country offices, and independent consultancies (Table S2). Data collection teams interviewed key personnel of the water supply systems (i.e., managing directors, water quality managers, operations staff) in their local language. For approximately half of the sites, data collectors received some form of training, while the rest relied solely on the data collection forms for guidance.

Two data collection forms were used at each site (Figure S2): (1) an impact assessment form to collect data on the 36 indicators listed in Table 2 and (2) an audit form to evaluate the WSP level of development and stage of implementation, used only during follow-up data collection. Two versions of the audit form were available: one for urban and one for rural systems. The audit forms used are included in Appendix B of *A practical guide to auditing water safety plans* [22]. Sites were defined as rural or urban by country institutions themselves and definitions varied between countries. In addition to quantitative data, qualitative data were collected using the impact assessment form through (1) explanations provided by data collectors regarding quantitative responses and (2) open-ended questions to interviewees about the impacts and challenges of WSPs.

### 2.4. Data Processing and Analysis

Data from the forms were collated and cleaned in Excel. Inconsistencies between data provided and qualitative explanations were checked, as well as those between baseline and follow-up data, and clarifications were sought with country officials and data collection teams. Data that were missing, indicated to be only estimates, clear outliers, or obviously incorrect calculations were excluded. To evaluate the reliability of the indicators involving subjective scoring (operations and management (O1b) and equity (E1a), Table 2), an Aquaya staff member performed an independent scoring for 25% of sites using the available qualitative data. To assess how the operational impact assessment framework “performed,” the quality and availability of the data collected for each of the 36 indicators were analyzed. To assess data quality, each indicator was examined in depth to see whether data were presented in a consistent way before and after WSP implementation and between countries, reflected adequate understanding of the question, and could be verified using contextual information. This allowed for the identification of indicators that were prone to confusion, challenging to quantify, or inappropriate in specific settings. Indicators for which data errors or inconsistencies were common were defined as being of poor quality, and indicators that were missing in over 75% of sites were defined as insufficiently available.

We selected 25 sites representing all 12 countries for qualitative data analysis based on the following criteria: (1) inclusion of all countries (at least one and not more than three sites per country); (2) availability of qualitative data in the data collection form and (3) representation (e.g., urban and rural settings, range of water supply systems, and range of population served). Qualitative data analysis was performed using the NVivo software [23]. Baseline and follow-up data were inductively coded using codes such as “complaints”, “testing”, and “lack of documentation”. Coding queries and intersections between codes then allowed themes to emerge, such as “water quantity issues” or “operational costs”.

Statistical before-after comparisons were performed for indicators meeting two inclusion criteria: (1) both baseline and follow-up data were available from at least 25% of sites and (2) there were no substantial concerns with data quality. Out of 36 indicators, twenty did not meet these inclusion criteria (see Section 3.2). In addition, two indicators did not lend themselves to before-after comparisons because they only applied to follow-up (O1a and F3a). Statistical analysis was thus performed on fourteen indicators. The statistical significance of before-after differences was analyzed using the paired Wilcox rank-sum test for continuous indicators or the chi-square test for binary indicators. Correlations between audit scores and impact/outcome indicators were also investigated.

### 2.5. Ethics Statement

Written government approval for the study was obtained from Bangladesh, Cambodia, Lao PDR, Mongolia, Philippines, Sri Lanka, and Vanuatu. Government approval was implicit for Bhutan, Cook Islands, Nepal, Timor-Leste, and Samoa, where data collection was done by government agencies. The study protocol was submitted to the Western Institutional Review Board (WIRB, wirb.com, Olympia, WA, USA) for ethical review and received a determination of exemption from full review under 45 CFR §46.101(b)(2) of the Common Rule in the USA.

## 3. Results

### 3.1. WSP Audit Scores

**Figure 4:**
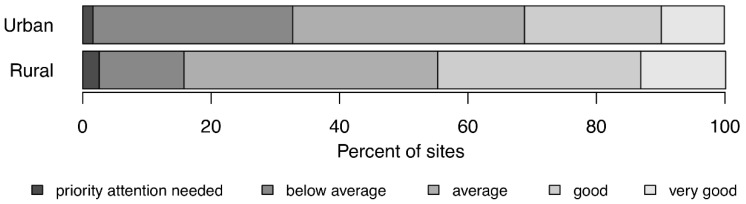
Distribution of qualitative audit scores assessing the quality of WSP implementation.

The WSP audit scores varied substantially between sites (Figure 4). No statistically significant relationships were found between audit scores and WSP age or population served (*p* > 0.05). The audit scores showed similar distributions for urban and rural sites, with the “average” category being the largest for both groups (Figure 4). The study generally did not find statistically significant relationships between audit scores and the outcomes or impacts measured. The absence of direct correlation between WSP implementation quality and outcomes/impacts suggests that WSP benefits result from a complex interplay of factors not limited to implementation rigor.

### 3.2. Evaluation Process: Analysis of the Indicators

Overall, 20 indicators suffered from substantial data quality issues (18), insufficient data availability (8), or both (6) (Table 2). Data were rarely available (i.e., in less than 25% of sites) for knowledge indicators (I2a-b), pressure (W1c), disinfectant residual compliance (W2f), “other” water quality parameters (W2g), consumer satisfaction (W3b), number of consumer complaints (W3d), and diarrheal incidence (H1c) (Table 2). In addition, financial (F1a-b, F2a-b, F3b), knowledge (I2a-b), equity (E1a), pressure (W1c), “other” water quality parameters (W2g), number of consumer complaints (W3d), health (H1a-c) and policy (P1a-b, P2a-b) indicators suffered from poor data quality (Table 2). With respect to financial indicators, the concerns included calculation mistakes, incomplete operating costs (missing line items), and misinterpretation of “revenue” (expected versus actually collected). For knowledge indicators, there were instances where different water system staff were interviewed at baseline and follow-up, limiting the ability to rigorously assess progress in knowledge. For equity indicators, the qualitative data revealed variability in the interpretation of “disadvantaged groups” and “explicit considerations of equity” between data collectors. For pressure, estimates were often provided, as opposed to actual measurements. Data on “other” water quality parameters and customer complaints suffered from inconsistent reporting, either between baseline and follow-up or between sites. With respect to health indicators, the primary concern was that information on disease incidence was collected from hospitals whose service areas did not necessarily align with that of water systems. Policy indicators were undermined by different interpretations of risk management between countries.

The remaining 16 indicators had both sufficient data availability and quality for analysis. Data from 14 of these indicators were quantitative and suitable for statistical analysis: operations and management (O1b), stakeholder communication (I1a-c), service (W1a-b and W1d), water quality (W2a-e), and consumer feedback (W3a and W3c) indicators. The two qualitative indicators—infrastructure improvements (O1a) and financial support received (F3a)—were assessed at follow-up only and were therefore considered outside of the statistical analysis.

Finally, for the two indicators relying on subjective scoring—level of operations and management practices (O1b) and equity (E1a)—scores assigned by data collection teams and by Aquaya staff were not significantly different (*p* > 0.1, except for 3 sub-indicators for which the discrepancies were small, Table S3), suggesting that such subjective indicators do not constitute a weakness of the impact assessment framework.

Disparities were observed in data availability between urban and rural sites, especially for financial and water supply indicators. Many rural sites did not have data on operating costs and revenue (F1a-b, F2a-b) because they distributed water for free, relied on volunteer management, or did not hold official records. For example, a rural site in Timor Leste reported: “*Two technicians [are] working as volunteers for operation and for maintenance with the support of [donor] and community ... There is no system for collecting revenue from consumers as of now*”. A rural site in Samoa stated: “*The caretaker … is not paid on monetary terms but by other means*”. According to a rural site in Mongolia, “*Revenue collected from users is spent on incentives and salaries for the well operator. However, no records are kept*”. Similarly, indicators on water quality testing and compliance (W2a-e) were, on average, available at only 57% of rural sites (compared to 76% of urban sites), due to a lack of water quality testing at many sites. As a rural site in Mongolia stated, “*Laboratory testing is expensive; therefore, testing cannot be performed regularly*”. In Nepal, a rural site reported not having its own test kit: “*[We are] dependent on [the] District headquarter test kit and it seems [a] little bit impossible [to do] frequent tests*”. In Lao PDR, a rural site stated: “*Someone else [has] collected water samples and tested, but not sure whether it was for microbial indicators or not. No records of water quality testing results [are] available at the village*”. In addition, no rural site could provide data on unaccounted-for water (W1d) at both baseline and follow-up, primarily due to the absence of bulk and/or consumer meters. These results illustrate the challenges of evaluating lower-capacity sites where data are less available than at higher-capacity sites. These challenges were even more apparent for the four non-piped water sources in Cambodia, where data were available for only five performance indicators (O1b, I1a-c, and W2a).

### 3.3. Outcomes and Impacts of WSPs in the Asia-Pacific Region

The vast majority of the sites (83%, 54 urban and 28 rural) reported infrastructure improvements as a direct result of WSP implementation (O1a). These improvements targeted various stages of the water supply system, including the catchment/source (e.g., toilet construction, flood protection), the treatment plant (e.g., capacity increase, automatic chlorination, new reservoirs), the distribution system (e.g., network expansion, new pumps, meter installation), the energy supply (e.g., purchase of generators), and monitoring activities (e.g., laboratory construction). Qualitative data indicated that infrastructure improvements were generally focused on water quality rather than water quantity. Several factors were cited as having facilitated these improvements, including recommendations by WSP teams, the enhanced authority of water quality divisions, and changes in management mindsets with respect to water safety risks. In addition, over a third of the sites (37) reported that WSP implementation was linked to financial support from donors or NGOs (other than DFAT) (F3a).

Aggregated results for the 14 quantitative performance indicators that met the inclusion criteria are presented in Table 3. Between baseline and follow-up, statistically significant improvements were observed in operations and management practices (O1b), the number of water safety-related meetings (I1a, I1b, I1c), unaccounted-for water (W1d), water quality testing activities (W2a, W2c, W2e), and monitoring of consumer satisfaction (W3a, W3c) (Table 3). The median score for the level of operations and management practices increased from 9% to 44% (*p* < 0.01, *n* = 93) (Table 3), showing greater attention to proactive risk management practices. The proportion of sites reporting internal, external, and consumer water safety meetings increased from 16% to 60% (*p <* 0.01, *n* = 92), 25% to 48% (*p <* 0.01, *n* = 92), and 16% to 53% (*p <* 0.01, *n* = 85), respectively (Table 3). The median level of unaccounted-for water (UFW) decreased from 25% to 20% (*p =* 0.01, *n* = 30) (Table 3). The median number of microbial, turbidity, and disinfectant residual tests performed increased from 3 to 12 (*p <* 0.01, *n* = 89), 0 to 4 (*p <* 0.01, *n* = 87), and 0 to 10 tests per year (*p <* 0.01, *n* = 74), respectively (Table 3). Between baseline and follow-up, the number of sites that conducted consumer satisfaction surveys and kept records of consumer complaints increased from 13% to 33% (*p <* 0.01, *n* = 92), and 41% to 61% (*p <* 0.01, *n* = 92), respectively (Table 3). In contrast, there were no significant changes in service continuity (W1a), service coverage (W1b), or in the compliance of test results with national standards (W2b and W2d) (Table 3). For the latter, the absence of observed changes may be due to the unavailability of data in a large number of sites and to reports of 100% compliance at baseline for the remaining sites (Table 3). All improvements in WSP outcomes and impacts observed in this study are summarized in Table 4.

The qualitative data indicated that knowledge and training obtained by water system staff through WSP implementation increased their attention to water quality, and that increased testing in turn improved their understanding of the system and their motivation to ensure water quality. Qualitative data also indicated that consumer complaints at follow-up were more often focused on water quantity (e.g., reliability, pressure) than on water quality.

The qualitative data analysis also revealed a number of challenges in the implementation of WSPs. Many sites reported that they were unable to implement risk mitigation measures due to financial constraints. Several sites noted that staff transfers or insufficient staffing levels impeded WSP implementation. Although statistically significant improvements were observed in operations and management practices (O1b), a number of sites reported persisting challenges related to operations and management, reflected in a lack of written procedures, poor record keeping, reactive (as opposed to proactive) maintenance, and discrepancies between planned and actual water quality testing. Some sites noted challenges in convening water safety meetings, especially when WSP team members were from different local government departments. These accounts are consistent with the generally low levels of operations and management practices (O1b) and number of meetings (I1a-b-c) reported in Table 3.

## 4. Discussion

### 4.1. How to Improve the WSP Impact Evaluation Process

This study, which was the first to use a comprehensive impact assessment framework to assess the effectiveness of WSPs across multiple countries and regions, identified a number of challenges relating to performance indicators, data collection, and the broader approach to WSP impact assessments. This section provides suggestions to address these challenges and better adapt the evaluation process to its context and objective. A revised impact assessment framework will be developed by WHO based in part on these suggestions and published as a separate document. 

Based on the experiences with data collection across a wide range of sites, the 36 indicators have been categorized into four groups according to recommendations for future impact assessments (Table 2):

- Retain without changes. Indicators are important and reliable data were easily collected.
- Retain but modify to standardize answers and avoid calculation mistakes. Indicators are important but were associated with data quality and/or availability challenges that can be easily overcome.
- Retention requires further consideration. Indicators were associated with significant data quality and/or availability challenges that may be difficult to overcome (except at higher-capacity sites). If retained, modifications will be needed.
- Do not retain. Indicators are not core to the WSP process, are redundant and/or are not sufficiently important to warrant addressing data quality and/or availability challenges experienced.

Indicators in categories B and C should only be retained when they can yield standardized and comparable data across sites. To achieve this, data collection methods should take into consideration the capacity of water systems and provide step-by-step guidance where needed. Finally, although knowledge and health indicators were particularly challenging to quantify and did not yield useful results, they represent core target aspects of WSPs. Therefore, more robust and standardized methods to measure these indicators are needed so that these indicators can be included in future impact assessment frameworks.

The results also suggest that some indicators in categories A and B may need different data collection methods to ensure better data quality. Firstly, three indicators—service continuity (W1a), consumer satisfaction (W3b), and diarrheal incidence (H1c)—would likely yield more accurate data if evaluated through household surveys as opposed to questionnaires to the water supplier (W1a and W3b) and review of existing household data (H1c). In addition, if diarrheal incidence is to be evaluated, it needs to be appropriately sampled and statistically powered [24]. Secondly, for low-capacity systems that lack data, external measurements of water quality may be needed (e.g., to establish baseline data). For these water systems, data collectors may also need to clarify and assist with financial calculations when information on revenue or cost recovery is not readily available.

More generally, this study revealed two important challenges inherent to WSP impact assessments. Firstly, a relationship between the performance of a water system and the availability of data was observed. The sites for which data were most available were higher-capacity sites with high indicator levels at baseline. By contrast, lower-capacity sites with the most room for improvement often lacked reliable data at baseline, limiting the ability to detect changes. Therefore, to assess the extent to which WSPs can improve lower-capacity systems, data collection methods tailored to such systems (such as those recommended above for water quality and financial issues) are needed. Secondly, the diversity in system types (piped/point sources, urban/rural, small/large) and WSP age can be expected to lead to heterogeneities in the results achieved through WSP implementation. For example, a favorable WSP outcome for a low-capacity rural system may have been an increase in microbial water testing activities due to prioritization of monitoring practices, whereas a high-capacity urban system may have reduced the number of microbial tests due to improved efficiencies and risk management procedures. Looking for average effects in the entire sample would not reveal such case-specific outcomes. Therefore, limiting sample heterogeneity or selecting a sample size large enough to investigate sub-categories is recommended.

The design of future impact assessments should be selected according to their context and objective. Table 5 describes study designs falling under two categories: controlled and uncontrolled. On one hand, research studies aiming to rigorously determine the impacts of WSPs will require a “control” group comparable to the “intervention” group, as well as consistent and independent data collection. The intervention and control groups should be either randomly selected (randomized controlled trial) or methodically matched on a variety of relevant factors such as water system size, geographic setting, and/or revenue (matched controls trial). Such methodical study designs will ensure that observed changes in indicators can be attributed to WSP implementation, and will thus increase the generalizability and policy-relevance of the findings. On the other hand, country- or site-level monitoring and evaluation do not necessarily require controlled study designs. Although they cannot rigorously establish causality between WSP implementation and changes in indicators, uncontrolled designs such as before-after comparisons can be valuable to support advocacy and encourage better monitoring practices. Where resources are available, a before-after comparison can be made more robust by using historical data on indicators (as opposed to single “before” and “after” data points) and looking for changes in trends that coincide with WSP implementation (interrupted time series design). For all study designs, it is important to note that external validity (i.e., the generalizability of findings to an entire country or region) will depend on the extent to which study sites are representative of water systems in this country or region. Evaluators should thus select study sites using deliberate criteria to ensure representativeness. Random selection of water systems within the country or region of interest would ensure the highest degree of representativeness.

Finally, we recommend that future impact assessments include WSP audits as part of the methodology. Audits help ensure that interventions are implemented sufficiently well, i.e., that key intervention outputs are delivered, without which further assessments of outcomes and impacts may not be relevant. Audits can also help identify aspects of WSP implementation that need to be improved or adapted, which is key for programmatic improvements [25].

### 4.2. Achievements of WSPs in the Asia-Pacific Region

Despite the challenges described above, this study provided important insights into the implementation of WSPs in the Asia-Pacific region. A number of improvements in management procedures (levels of operations and management practices, number of water safety-related meetings, water quality testing activities, monitoring of consumer complaints), infrastructure, and finance were associated with WSP implementation. Given that most WSPs were only 1–3 years old, these indicators were the most likely to show significant improvements. The study also identified changes that are expected to occur over longer time periods related to water service delivery, including a reduction in unaccounted-for water. Although a previous literature review found limited evidence for institutional effects driven by WSPs [16], this study did identify institutional changes, such as an increase in the number of water-safety meetings and a greater attention to water quality reflected in the qualitative data. However, the qualitative data identified a number of challenges in the implementation of WSPs, including financial barriers and obstacles to the improvement of management procedures. This study also found that infrastructure improvements prioritized water quality rather than quantity while consumer complaints were primarily focused on water quantity, highlighting the importance of balancing water quality and quantity considerations through water safety planning.

### 4.3. Study Limitations

This impact assessment had several limitations. Firstly, the study design did not include a comparison (“control”) group; therefore, although the observed changes were associated with WSPs, it is not possible to attribute causality to WSP implementation. Secondly, the site selection was not random, which may have led to the selection of “best-case” scenarios. Thirdly, data were frequently unavailable; therefore many relationships could not be tested. For example, the data did not allow us to investigate differences in outcomes and impacts between small and large, or between low- and high-capacity systems. Fourthly, the wide range of training and experience among data collectors led to issues with data consistency and quality. However, the analysts have attempted to address some of these limitations by carefully cleaning the data and checking for inconsistencies, and by only analyzing trends for indicators without data quality concerns and available at >25% of sites. Lastly, baseline data collection was retrospective at approximately two-thirds of the sites, which reduced the ability to measure knowledge indicators and may have led to recall errors. These limitations should be carefully addressed in future WSP impact assessments. Specifically, it is recommended to improve training of data collectors and assistance to lower-capacity water systems to collect and report data. Finally, this study showed the importance of collecting qualitative data to substantiate the quantitative indicators.

## 5. Conclusions

This study was the first attempt to conduct a comprehensive impact evaluation of WSPs on a regional scale. A number of positive outcomes and impacts from WSP development and implementation were identified, as were challenges. In addition, substantial heterogeneities between sites were observed, especially in data availability and record keeping. Therefore, increased efforts by water suppliers and regulators to improve data collection and recording practices are needed.

The study found that the process of assessing the impact of WSPs can be improved, and a number of suggestions have been made to refine the study design and the data collection process. A revised impact assessment framework will be developed by WHO based in part on these suggestions and published as a separate document. In addition, it is recommended that future research investigate the drivers and barriers for WSP success. More systematic monitoring of WSPs is needed to improve their implementation process, guide their scale-up, and build political support around them.

## Figures and Tables

**Table 1:** Number of sites that participated in the WSP impact assessment by country, type of water system, context (urban/rural), and WSP age.

Country	Total Sites	Type of System			Context		Age	
		Piped Systems		Point Sources	Urban	Rural	<2 years	>2 years
		Utility/LGU ^a^	Community-Managed ^b^					
Bangladesh	10	8	2	0	8	2	7	3
Bhutan	13	6	7 ^b^	0	7	6	10	3
Cambodia	8	4	0	4 ^c^	4	4	0	8
Cook Islands	1	1	0	0	1	0	0	1
Lao PDR	5	3	0	2 ^c^	3	2	1	4
Mongolia	8	7	0	1 ^c^	3	5	5	3
Nepal	15	1	14	0	11	4	9	6
Philippines	15	12	0	3 ^d^	8	7	11	4
Samoa	1	0	1	0	0	1	1	0
Sri Lanka	10	10	0	0	10	0	4	6
Timor-Leste	10	6	4	0	6	4	10	0
Vanuatu	3	0	3	0	0	3	3	0
Total	99	58	31	10	60	39	61	38

^a^ Private or public utility or Local Government Unit (LGU); ^b^ three systems managed by schools; ^c^ groundwater sources; ^d^ water refilling stations.

**Table 2:** Outcome and impact indicators (all are site-level, except policy indicators, which are country-level). For each indicator, data availability (at both baseline and follow-up), data quality, and suggestions for revising the impact assessment framework are reported. On the basis of these recommendations and other inputs, WHO will publish a separate document detailing revised indicators and associated data collection guidance.

Code	Indicator	Data Format	Availability (% of Sites) ^5^	Data Quality	Category ^6^	Comments on Suggested Revisions
**O**	**Operational Outcomes**					
O1a	Infrastructure change as a result of WSP ^1^	Y/N, description	95	Good	A	Retain
O1b	Level of operations and management practices	Score of 8–40 (score of 1–5 each)	93	Good		
	(1) Operational monitoring plan				A	Retain
	(2) Compliance monitoring plan				A	Retain
	(3) Consumer satisfaction monitoring				D	Exclude because redundant with W3b
	(4) Standard operating procedures				A	Retain
	(5) Emergency response plan				A	Retain
	(6) Operator or caretaker training programs				A	Retain
	(7) Consumer education programs				D	Exclude because redundant with I1c
	(8) Equipment maintenance/calibration schedules				C	Reconsider including as addressing such maintenance schedules is not emphasized in the WSP process
**F**	**Financial Outcomes**					
F1a	Operating costs per unit water ^2^	$/m^3^	72	Poor	D	Exclude to simplify; revenue to cost ratio will suffice
F1b	Operating costs per population ^2^	$/pop	71	Poor	D	Exclude to simplify; revenue to cost ratio will suffice
F2a	Revenue per population ^2^	$/pop	71	Poor	D	Exclude to simplify; revenue to cost ratio will suffice
F2b	Revenue to cost ratio ^2^	%	66	Poor	B	Retain but provide a step-by-step calculation guide to avoid mistakes and standardize the definitions of operating costs and revenue
F3a	Financial support as a direct result of WSP ^1^	Y/N, description	89	Good	A	Retain
F3b	Funds from government for water supply	$/description	59	Poor	B	Retain but combine with indicator F3a and provide more guidance to clarify indicator and to improve reliability of data
**I**	**Institutional Outcomes**					
I1a	Internal water safety meetings ^2^	Number	92	Good	A	Retain
I1b	External water safety meetings ^2^	Number	92	Good	A	Retain
I1c	Consumer water safety trainings ^2^	Number	85	Good	A	Retain
I2a	Understanding of system ^3^	Score of 5–25	19	Poor	C	Reconsider including due to lack of meaningful measurements (unless a more effective and systematic measurement approach can be designed)
I2b	Understanding of hazards ^3^	Number	19	Poor	C	
**E**	**Equity Outcomes**					
E1a	Equity ^4^	Score of 6–30 (score of 1–5 each)	88	Poor	C	Reconsider including due to widespread misinterpretation until explicit consideration of equity through the WSP process is widely promoted
	(1) Participation					
	(2) Groups identified and documented					
	(3) Hazards/issues prioritized					
	(4) Improvements benefit equitably					
	(5) Monitoring data disaggregated					
	(6) Emergency response and communication programs reflect needs					
**W**	**Water Supply Impact**					
W1a	Continuity	Hours/week	93	Good	B	Retain but consider refining guidance to avoid rough estimates of continuity
W1b	Service coverage	%	76	Good	C	Reconsider including as expanding service coverage is often not a core priority or key outcome of WSPs
W1c	Pressure	atm/bar/m	22	Poor	C	Reconsider including due to data quality concerns (variable measurement methods and tendency to provide rough estimates)
W1d	Unaccounted-for Water (UFW)	%	30	Good	B	Retain but revise guidance to better distinguish between UFW and non-revenue water (NRW)
W2a	Microbial tests ^2^	Number	89	Good	A	Retain
W2b	Microbial compliance ^2^	%	60	Good	A	Retain
W2c	Turbidity tests ^2^	Number	87	Good	A	Retain
W2d	Turbidity compliance ^2^	%	37	Good	A	Retain
W2e	Disinfectant residual tests ^2^	Number	74	Good	A	Retain
W2f	Disinfectant compliance ^2^	%	21	Good	A	Retain
W2g	Other water quality parameter compliance ^2^	%, description	0	Poor	B	Retain but standardize list of parameters and formatting
W3a	Consumer satisfaction surveys conducted	Y/N	92	Good	A	Retain
W3b	Consumers satisfied ^2^	%	10	Good	B	Retain but consider recommending a household survey where suppliers do not have standardized data
W3c	Consumer complaint records kept	Y/N	92	Good	A	Retain
W3d	Number of consumer complaints ^2^	%	22	Poor	B	Retain but standardize reporting
**H**	**Health Impact**					
H1a	Cases of diarrhea ^2^	Number	43	Poor	B	Retain but revise guidance to highlight/address common discrepancies between health center and WSP coverage areas
H1b	Other water-related illnesses ^2^	Number	31	Poor	B	Retain but revise guidance to highlight/address common discrepancies between health center and WSP coverage areas and combine with indicator H1a
H1c	Diarrheal incidence ^2^	%	5	Poor	B	Retain but change to primary household data collection rather than review of existing household data available
**P**	**Policy Outcomes**					
P1a	Proactive water quality risk management approaches are/were included in formal water sector policies or regulations at time of follow-up assessment	Y/N, description	92	Poor	B	Retain but provide a standardized definition of risk management
P1b	Activity to develop or revise national drinking water quality standards has been undertaken	Y/N, description	92	Poor	D	Exclude because difficult to obtain information in a standardized and meaningful way and link to WSP implementation
P2a	Proactive water quality risk management approaches have been adopted by other water-sector stakeholders (e.g., NGOs, UNICEF)	Y/N, description	83	Poor	D	Exclude because difficult to obtain information in a standardized and meaningful way
P2b	Proactive water quality risk management approaches are promoted in national or sub-national programs	Y/N, description	83	Poor	C	Reconsider including this indicator reflects drivers of WSPs as opposed to outcomes

^1^ Only asked at follow-up; ^2^ cumulative value over the 12-month period before data collection; ^3^ only asked if baseline data collection was prospective; ^4^ all elements refer to women and/or disadvantaged groups; ^5^ except for Policy Outcomes, where the unit is “% of countries”; ^6^ suggestions regarding each indicator fall into four categories: A. Retain without changes. Indicators are important and reliable data were easily collected; B. Retain but modify to standardize answers and avoid calculation mistakes. Indicators are important but were associated with data quality and/or availability challenges that can be easily overcome; C. Retention requires further consideration. Indicators were associated with significant data quality and/or availability challenges that may be difficult to overcome (except at higher capacity sites). If retained, modifications will be needed; D. Do not retain. Indicators are not core to the WSP process, are redundant and/or are not sufficiently important to warrant addressing data quality and/or availability challenges experienced.

**Table 3:** Comparisons of WSP outcome and impact indicators between baseline and follow-up. The analysis includes the number of sites (*n*), the percentage of sites reporting any given activity, median values across all sites at baseline and follow-up, and the statistical significance of the change between baseline and follow-up (*p*-value). *p*-values were determined using the paired Wilcox rank-sum test for all except the binary W3a and W3c indicators, which were determined using the chi-squared test. Results for each indicator, except O1b, are reported for 12-month periods.

Code	Indicator	*n*	Unit	% of Sites		Median Values		*p*-Values
				Base-Line	Follow-Up	Base-Line	Follow-Up	
Operational Outcomes								
O1a	Infrastructure changes due to WSP	95	yes/no	-	86	-	-	-
O1b	Level of operations and management practices	93	%	-	-	9	44	<0.01
Financial Outcomes								
F3a	Financial support due to WSP	89	yes/no	-	42	-	-	-
Institutional Outcomes								
I1a	Internal meetings	92	number	16	60	0	2	<0.01
I1b	External water safety meetings	92	number	25	48	0	0	<0.01
I1c	Consumer water safety trainings	85	number	16	53	0	1	<0.01
Water Supply Impact								
W1a	Continuity	93	h/week	34 ^a^	37 ^a^	97	104	0.59
W1b	Service coverage	76	%	-	-	85	81	0.75
W1d	Unaccounted-for water (UFW)	30	%	-	-	25	20	0.01
W2a	Microbial tests	89	number	73	85	3	12	<0.01
W2b	Microbial compliance	60	%	-	-	99	98	0.24
W2c	Turbidity tests	87	number	45	70	0	4	<0.01
W2d	Turbidity compliance	37	%	-	-	100	100	0.5
W2e	Disinfectant residual tests	74	number	39	57	0	10	<0.01
W3a	Consumer satisfaction surveys	92	%	13	33	-	-	<0.01
W3c	Consumer complaint records	92	%	41	61	-	-	<0.01

^a^ Sites reporting continuous supply.

**Table 4:** Summary of observed WSP outcomes and impacts.

Indicators	Observed WSP Outcomes and Impacts	% of Sites Showing Improvements ^1^ (and Number of Countries)
O1a	Infrastructure improvements	86% (10 countries)
O1b	Improvement in operation and management	95% (12 countries)
F3a	Leveraging of donor funds	39% (9 countries)
I1a, b, c	Increased stakeholder communication and collaboration	66% (10 countries)
W1d	Reduction in unaccounted-for water (UFW)	21% (7 countries)
W2a, c, e	Increased water quality testing	65% (11 countries)
W3a, c	Increased monitoring of consumer satisfaction	33% (11 countries)

^1^ For groups of indicators, the % of sites showing improvements in at least one indicator are reported.

**Table 5:** Possible study designs for future WSP impact assessments, with advantages and challenges.

	**Uncontrolled Study Designs**		**Controlled Study Designs**	
	*Context: Site- or Country-Level Monitoring and Evaluation*		*Context: Research and Rigorous Impact Assessments*	
	**Before-after Comparison**	**Interrupted Time Series**	**Matched Controls**	**Randomized Controlled Trial**
Control group	No control group; for each site, relevant indicators are compared before and after WSP implementation	No control group; for each site, historical time series of relevant indicators are investigated to detect potential changes in slope coinciding with WSP implementation	Before WSP implementation, sites are manually assigned to a “control” or “intervention” group by matching a number of selected parameters between the two groups (e.g., system size, age, revenue, geographic setting)	Before WSP implementation, sites are randomly assigned to a “control” or “intervention” group. The randomization ensures that all possible confounding factors are equally distributed amongst the two groups.
WSP implementation	To all sites	To all sites	Only to “intervention” group	Only to “intervention” group
Data needed	Baseline and follow-up data	Historical data (pre- and post-WSP) on all relevant indicators (i.e., time series, not just baseline and follow-up data)	Inventory of all eligible study sites with data on parameters for matching Baseline and follow-up data	Inventory of all eligible study sites, ideally with data on some key parameters to confirm comparability between intervention and control groups Baseline and follow-up data
Advantages	Simplest study design (does not require a control group and only two data points per indicator: before and after) Results can be valuable for national advocacy and to encourage better monitoring/data collection practices Two rounds of data collection	Does not require a control group Provides more confidence than a simple before-after comparison that the changes observed may be associated with WSP implementation	A rigorous study design to examine associations between WSP implementation and outcomes/impacts, as long as all key parameters potentially affecting a water system’s performance (i.e., confounding factors) are used for matching Two rounds of data collection	The only study design able to establish causality, i.e., the differences between the control and intervention groups can be attributed to WSP implementation because confounding factors are equally distributed amongst the two groups Two rounds of data collection
Challenges and limitation	Causality cannot be established from a simple before-after comparison, i.e., the changes observed cannot be attributed to WSP implementation	Limitations in establishing causality (i.e., the change in slope observed cannot be rigorously attributed to WSP implementation) Multiple (>2) rounds of data collection Difficult to obtain time series of all relevant indicators, especially in low-capacity sites that do not keep rigorous records. Where available data are limited, data collection could be limited to those indicators that are most likely to show changes (as identified by prior rigorous impact assessments conducted at other sites)	Difficult to obtain data on matching parameters, especially for small water systems Risk that confounding factors may be unevenly distributed between the two groups (especially if an insufficient number of parameters are selected for matching), limiting ability to establish causality	Randomizing WSP implementation may cause ethical concerns or political frictions. To mitigate these, WSPs could be implemented in the control group at the end of data collection (i.e., staggered implementation).

## Supplementary Materials

The following are available online at http://www.mdpi.com/1660-4601/15/6/1223/s1, Figure S1: Description of the WSP process, Figure S2: Flowchart of data collection and analysis process, Table S1: Number of sites participating in the impact assessment in each country and criteria used for their selection, Table S2: Description of data collection teams in each country, training processes, dates of baseline and follow-up data collection, and proportion of sites where baseline data were collected retrospectively, Table S3: Comparison of data collectors’ scores with independently assigned scores by Aquaya staff.
