# Supplementary material for: Measuring the Impacts of Water Safety Plans in the Asia-Pacific Region
Source: Int J Environ Res Public Health. 2018 Jun 10;15(6):1223. doi: 10.3390/ijerph15061223 (PMC6025033; doi:10.3390/ijerph15061223)
Supplement: Supplementary file 1 [file ijerph-15-01223-s001.pdf]

Table S1. Number of sites participating in the impact assessment in each country and criteria used for their selection. Institutions responsible for selecting study sites varied between countries and included national-level government agencies (e.g. departments/ministries of health and water), national water suppliers, heads of local government units, and often WHO country or regional staff.

Country	Number of sites	Criteria for selecting WSP sites
Bangladesh	10	Targeted a distribution across new urban WSPs, new rural WSPs, and established WSPs
Bhutan	13	Based on geographical representation
Cambodia	8	Selected all WSP sites
Cook Islands	1	Based on data availability
Lao PDR	5	Well-implemented WSPs (provincial suppliers), logistics (small town water suppliers near these provincial suppliers), and oldest WSPs (rural)
Mongolia	8	Oldest WSPs (urban), pilot areas with well-trained and experienced WSP teams, or areas with need (water scarcity and water quality problems) (rural)
Nepal	15	Based on sites that fit the criteria received from WHO headquarters (a mix of urban and rural sites, established WSP sites and new WSP sites)
Philippines	15	Based on good data availability and older WSPs
Samoa	1	Site with a recently completed WSP
Sri Lanka	10	More mature WSPs
Timor-Leste	10	All pilot WSP sites were selected
Vanuatu	3	No information

Table S2. Description of data collection teams in each country, training processes, dates of baseline and follow-up data collection, and proportion of sites where baseline data were collected retrospectively.

Country	Data collection team organizations	Description of training process	Data collection dates		
			Baseline	Retro/ Total	Follow-up
Bangladesh	DPHE, ITN-BUET, PMID	Formal half-day training led by the NPO-WSHE and national WHO consultant	Nov 2014 - Mar 2015	9/10	Jan - Mar 2016
Bhutan	MOH, MoWHS, PHED, WHO, WSP Team, Dzongkhag Engineers, HA	No formal training/used forms as guidance	Sep 2014 - Apr 2015	3/13	Jan - June 2016
Cambodia	IITC, RUPP, WHO	No formal training/used forms as guidance	May 2015	8/8	Jan - Mar 2016

Cook Islands	WHO consultant	No formal training/ used forms as guidance	Feb 2015	1/1	Feb 2016
Lao PDR	CAWST, DHHP, DHOs, DHUP, DWS, MPWT, LPB, Provincial Offices, WHO	Field training for urban WSPs by WHO; no formal training for rural sites	Jan - Feb 2015	4/5	Feb - Apr 2016
Mongolia	General Authority of Specialized Inspection, MNUMS, WSSA – USUG	Formal, structured multi-day training	Jan - Feb 2015	5/8	Feb - May 2016
Nepal	DWSS, WHO, WSSD Udayapur	Data collection by master WSP trainer. No formal training; used forms as guidance.	Sep 2014 - Feb 2015	10/15	Feb - Mar 2016
Philippines	LCI Env Corp, DOH, LWUA	Organized by WPRO and supported by WHO SEARO via Skype/used forms as guidance	Feb 2015	4/15	Feb - Apr 2016
Samoa	WHO	No formal training/used forms as guidance.	Jan 2015	0/1	Mar 2016
Sri Lanka	NWSDB	One-day training by WHO consultant	Nov 2015 - Feb 2016	9/10	Dec 2015 - Apr 2016
Timor-Leste	WHO	Consultant trained remotely by WHO	Nov 2014 - Dec 2014	10/10	Feb 2016
Vanuatu	WHO, Provincial EHO	No information	Feb 2016	1/3	Feb 2016

Abbreviations: ITN-BUET: Bangladesh University of Engineering and Technology; PMID: Participatory Management Initiative for Development; DPHE: Department of Public Health Engineering; MoWHS: Ministry of Works and Human Settlement; PHED: Public Health Engineering Division; MOH: Ministry of Health; RUPP: Royal University of Phnom Penh; DHHP: Department of Hygiene and Health Promotion; DWS: Water Supply Division; MPWT: Ministry of Public Works and Transport; DHUP: Department of Housing and Urban Planning; CAWST: Centre for Affordable Water and Sanitation Technology; WSSA – USUG: Water Supply and Sewerage Authority; MNUMS: Mongolian National University of Medical Sciences; DWSS: - Department of Water Supply and Sanitation; DOH: Department of Health, LWUA: Local Water Utilities Administration; NWSDB: National Water Supply and Drainage Board; IITC: Institute of Technology Cambodia; DHOs: District Health Offices.

Table S3. Comparison of data collectors' scores with independently assigned scores by Aquaya staff. Differences are the independent researcher score subtracted from the country data collector score; positive values indicate that the data collector score was higher than the researcher score. p-values are the result of a paired t.test comparing the groups of scores. a – Median difference in scores.

	Indicator	Baseline			Follow-up		
		**n**	**Diff [a]**	***p***	**n**	**Diff [a]**	***p***
**O1b**	**Levels of operations and management practices**						
	1) Operational monitoring plan	25	-0.2	0.33	18	-0.2	0.33
	2) Compliance monitoring plan	25	0	1	16	0.1	0.75
	3) Consumer satisfaction monitoring	25	0	0.85	16	-0.1	0.77
	4) Standard operating procedures	25	-0.2	0.19	16	-0.9	<0.01
	5) Emergency response plan	24	0.2	0.5	16	0.1	0.72
	6) Operator or caretaker training	25	0.4	0.02	16	-0.1	0.33

	7) Consumer education programs	24	-0.2	0.38	17	-0.1	0.5
	8) Equipment maintenance/calibration	25	-0.1	0.65	16	-0.2	0.16
**E1a**	**Equity**						
	1) Participation	22	1.3	<0.01	11	0.5	0.11
	6) Emergency response and communication reflect needs	20	0.4	0.11	12	0	1

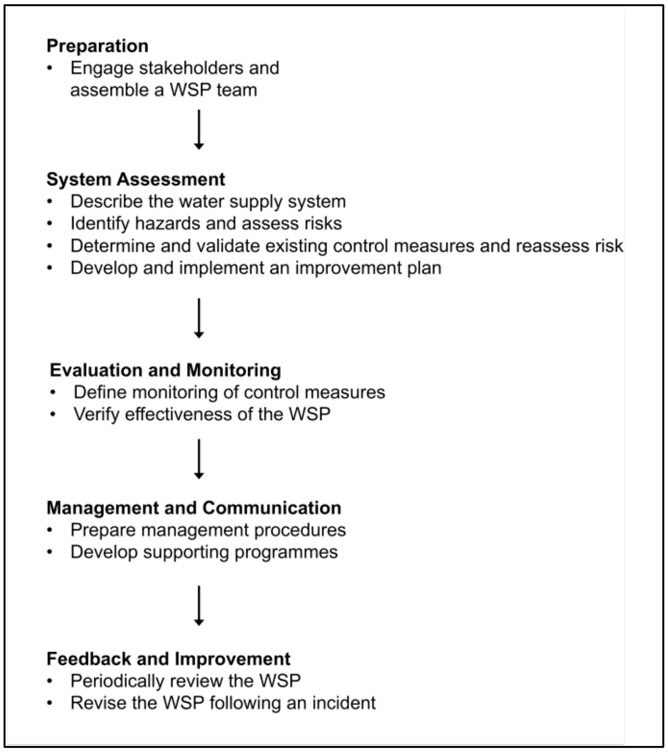

Figure S1: Steps of the WSP process.

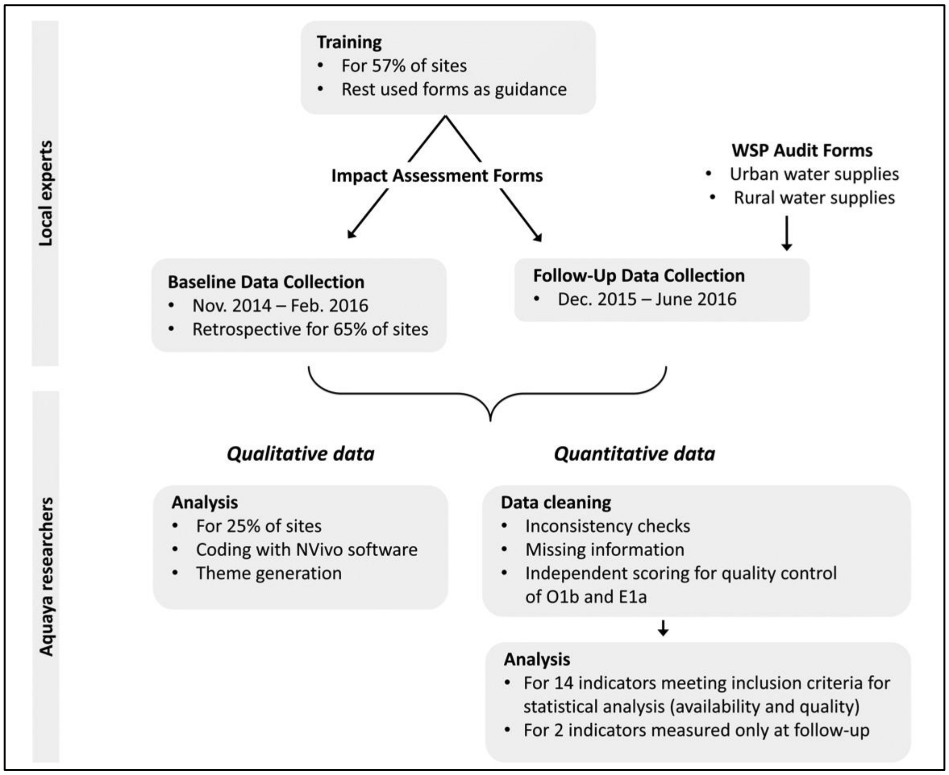

Figure S2. Flowchart of data collection and analysis process.
